# Discovery and translation of a target engagement marker for AMP-activated protein kinase (AMPK)

**DOI:** 10.1371/journal.pone.0197849

**Published:** 2018-05-25

**Authors:** Rolf Grempler, Michael Wolff, Eric Simon, Ramona Schmid, Claudia Eisele, Kathrin Rieber, Elke Fischer, Sonja Mettel, Ogsen Gabrielyan, Denis Delic, Gerd Luippold, Norbert Redeman

**Affiliations:** 1 Department of Translational Medicine and Clinical Pharmacology, Boehringer Ingelheim Pharma GmbH & Co. KG, Biberach an der Riss, Germany; 2 Department of Target Discovery Research, Boehringer Ingelheim Pharma GmbH & Co. KG, Biberach an der Riss, Germany; 3 Department of Cardio Metabolic Diseases Research, Boehringer Ingelheim Pharma GmbH & Co. KG, Biberach an der Riss, Germany; Univerzitet u Beogradu, SERBIA

## Abstract

**Background:**

Activation of the AMP-activated protein kinase (AMPK) is an attractive approach for the treatment of type 2 diabetes. AMPK activation reduces glucose levels in animal models of type 2 diabetes by increasing glucose uptake in skeletal muscles and reducing hepatic glucose production. Furthermore, AMPK activation ameliorates hepatic steatosis in animal models. For the clinical development of AMPK activators it is essential to have a reliable target engagement marker for appropriate dose finding and to support proof of clinical principle. While the activation of AMPK by quantification of the phosphorylation of AMPK at Thr^172^ in target tissues can be assessed pre-clinically, this is not feasible in clinical studies. Therefore, we attempted to identify and translate a peripheral target engagement biomarker downstream of AMPK activation for clinical use in blood samples.

**Methods:**

For pharmacological activation of AMPK, two AMPK activators were synthesized (compound 1 and 2). A compound with structural similarities but no pharmacological effect on AMPK phosphorylation was synthesized as negative control (compound 3). Whole blood from healthy volunteers was incubated with an AMPK activator for up to 6 hours and mRNA sequencing was performed. Additionally, human PBMCs were isolated to evaluate Thr^172^-phosphorylation of AMPK in Western blots. In order to enable identification of translatable biomarker candidates, blood samples from HanWistar rats treated for two weeks with an AMPK activator were also subjected to mRNA sequencing. Furthermore, concentration-response curves for four biomarker candidates were recorded in human blood samples using Nanostring nCounter technology. Finally, ZDF rats were treated with increasing doses of compound 2 for five weeks to investigate the glucose-lowering efficacy. To investigate changes of mRNA expression of two selected biomarker candidates in this ZDF rat study, qRT-PCR was performed.

**Results:**

Pharmacological activation of AMPK in human PBMCs revealed an increase in Thr^172^-phosphorylation of AMPK, confirming target engagement in these blood cells. RNA sequencing of human blood samples identified 608 deregulated genes after AMPK activation. Additionally, AMPK activation led to deregulation of 367 genes in whole blood from HanWistar rats which mapped to the respective human genes. 22 genes out of the intersection of genes deregulated in both species are proposed as potential translatable target engagement biomarker candidates. The most prominent genes were transmembrane glycoprotein NMB (GPNMB, osteoactivin), calcium-binding protein A9 (S100A9), peptidoglycan recognition protein (PGLYRP1) and Ras homolog gene family, member B (RHOB). Specificity for AMPK was shown by testing inactive compound 3 in HanWistar rats. The exposure-effect relationship for GPNMB was investigated in a subchronic study in diabetic ZDF rats. GPNMB showed a dose-dependent up-regulation both acutely and after subchronic dosing. GPNMB up-regulation correlated with an increased Thr^172^-phosphorylation of AMPK in liver and quadriceps muscle in rats.

**Conclusion:**

GPNMB has been identified as a translatable target engagement biomarker for use in clinical studies.

## Background

Activation of AMP-activated protein kinase (AMPK) could represent an attractive approach for the treatment of metabolic diseases such as obesity, type 2 diabetes and cardiovascular disease [1] 1. AMPK is a sensor of energy status that, when activated by metabolic stress, maintains cellular energy homeostasis by switching on catabolic pathways and switching off ATP-consuming processes [2]. AMPK is allosterically activated by AMP and synthetic compounds (e.g. A-769662) but also by upstream kinases that phosphorylate threonine 172 within the activation loop of the AMPK kinase domain [3]. AMPK activation by the AMP-analog AICAR reduces glucose levels in animal models of type 2 diabetes by increasing glucose uptake in skeletal muscles, reducing hepatic glucose production and reduces hepatic glucose output in type 2 diabetic patients [4,5]. Furthermore, AMPK activation ameliorates hepatic steatosis and leads to an increased formation of brown fat cells in animal models [6,7]. In addition, cardiac AMPK activation via the direct AMPK activator A-769662 attenuates myocardial ischemia-reperfusion injury in mice [8]. The antidiabetic drug metformin activates AMPK indirectly by increasing cellular AMP levels [9–11]. However, metformin has low potency for AMPK activation and lack specificity towards AMPK. Novel potent and selective AMPK activators were identified in the past years [3]. In this study two novel potent direct AMPK activators, compound 1 and 2 were used as pharmacological agents. For clinical application of AMPK activators it is essential to have a reliable target engagement marker to support dose-finding in patients. While the activation of AMPK through Thr^172^-phosphorylation in target tissues like skeletal muscle can be assessed pre-clinically, this is not feasible in clinical studies. Therefore, we attempted to identify and translate a target engagement biomarker for clinical use in blood samples downstream of the AMPK pathway.

## Material and methods

### AMPK compounds

Compound 1 represents example 163 of WO 2012/116145, Merck Sharp & Dohme Corp. Compound 2 represents another potent AMPK activator discovered at Boehringer Ingelheim Pharma GmbH & Co.KG. Compound 3, with structural similarities to compound 1 but no biological activity on AMPK, represents example 280 from WO 2014/031515, Merck Sharp & Dohme Corp. All compounds were prepared according to the experimental procedures incorporated in the patent applications and diluted in 100% DMSO. The two active compounds 1 and 2 were tested at 10μM on a standard selectivity panel of 68 receptors at Panlabs/Eurofins (S3 and S4 Tables). Items meeting criteria for significance of the vendor (> 50% inhibition or stimulation) were not found for both compounds. Compound 1 highest value was 28% inhibition and Compound 2 highest values was 25% inhibition. In addition the two active compounds were tested at 10μM on an Invitrogen kinase selectivity panel (S5 and S6 Tables). Items meeting the criteria of relevance of the vendor (>80% inhibition) were the following: Compound 1 showed only relevant inhibition for CHUK (IKKalpha) (1 kinase out of 264 tested). Compound 2 showed only relevant inhibition for MYLK (1 kinase out of 280 tested). The Invitrogen Kinase Selectivity screen is validated to measure inhibition of the respective kinases. The assays are not validated for measuring activation of the respective kinases, therefore the assays are not applicable to characterize activators.

### In vivo study with AMPK activators

For the discovery study, male HanWistar rats (RjHan:WI) with an age of 10–11 weeks (Janvier, France) were treated by oral gavage with 0.5% hydroxyethylcellulose as a vehicle or Compound 1 with 30 mg/kg twice daily over 2 weeks. At the end of the study, whole blood was sampled in RNAlater (Qiagen, Germany) from the animals 24 h after the last dosing and prepared for RNA sequencing.

For the validation study, male Zucker diabetic fatty ZDF rats (ZDF-Leprfa/Crl) with an age of 12–13 weeks (Charles River, Germany) were treated by oral gavage with 0.5% hydroxyethylcellulose as a vehicle or Compound 2 with 1, 3, 10, 30 mg/kg once daily over 30 days. In addition, lean male ZDF rats (ZDF-Lepr-lean(+/?)/ Crl) with an age of 12–13 weeks (Charles River, Germany) were treated by oral gavage with vehicle. The animals had free access to breeding diet (Kliba 2437, Provimi Kliba, Suisse) and tap water. On day 1 and day 24 of treatment whole blood was sampled sublingual in RNAprotect Animal Blood Tubes (Qiagen, Germany) from the animals before, 6 and 24 h after the last dosing and prepared for qRT-PCR. Blood glucose was measured on day 28 with a glucometer (OneTouch Ultra; LifeScan, Milpitas, CA, Coefficient of Variation <5%) from the tip of the tail under 2h-fasting condition and 2h after the compound dosing. At the end of the study animals were sacrificed using intraperitoneal injections of pentobarbital (250 mg/kg). Livers of the rats were removed for hepatic as well as skeletal muscle for phospho-Thr^172^-AMPK measurements.

#### Statistical analysis of subchronic study in diabetic ZDF rats

The *in vivo* data are presented as mean±SEM. Statistical comparisons were conducted by one-way ANOVA followed by Bonferroni post-tests. A *P* value < 0.05 was considered to show a statistically significant difference.

#### Concentration-response experiment in human whole blood using NanoString nCounter technology

The concentration-response experiment was performed on whole blood from four healthy volunteers. Samples were incubated for 6h at 37°C with 10μM, 3μM, 1μM, 300nM, 100nM, 30nM and 10nM compound 2 or DMSO followed by RNA extraction via QIAsymphony PAXgene Blood RNA Kit (Qiagen, Hilden, Germany) according to manufacturer’s instructions. Human RNA samples were diluted to a final concentration of 30 ng/μL. RNA expression was analysed using NanoString nCounter technology, a digital color-coded barcode technology (NanoString Technologies, Seattle, WA, U.S.A.) as described by the vendor and with the specific nCounter Gene Expression Custom CodeSet (XT_GX CodeSet and the nCounter Panel Plus/CodeSet Plus) synthesized by NanoString Technologies. 150 ng RNA were hybridized for 17 h at 65°C, applied to the nCounter Preparation Station and analyzed using the nCounter Digital Analyzer by counting the individual barcodes.

#### Data analysis of whole blood RNAExpression by nCounter NanostringTechnology

Gene expression values were extracted using nSolver analysis software v3.0 of Nanostring Technologies Inc. Reference “gene normalization method” was chosen to normalize gene expression values. ACTB, GAPDH, HPRT1 and POLR2A genes were selected as reference genes. To reduce the effect of variation of gene expression levels of reference genes geometric mean was utilized to calculate scaling factors for this normalization. Moreover using RNA-Free control “background noise” level was calculated and subtracted from normalized value. For more details see [12].

#### Specificity analysis via quantitative real-time PCR with rat blood

RNA samples were reverse-transcribed, using the High Capacity cDNA Reverse Transcription Kit (Life Technologies) according the manufacturer’s protocol. The expression of the RNAs was analysed using TaqMan^®^ Gene Expression MasterMix (Applied Biosystems) and rodent TaqMan^®^ Gene Expression Assays for Gpnmb (Rn00591060_m1), Pglyrp1 (Rn00584492_m1), Rhob (Rn04219494_s1) and S100A9 (Rn00585879_m1) (Applied Biosystems). Results were normalized to the expression of Tmcc2 (Rn01759254_m1) and Polr2a (Rn01752026_m1) which were identified as unchanged in the RNA sequencing experiment. The gene expression analysis was run on an SDS7900HT real time PCR system with a final amount of cDNA of 20ng per well; raw Ct values were calculated using the SDS software v2.4. RNAs with Ct values > 35/ undetermined were excluded. Fold-change of expression was calculated using the comparative Ct method (2^-ΔΔCt^).

#### Dose response analysis via quantitative real-time PCR with RNA of rat blood

Rat RNA samples were isolated using RNeasy^®^ Protect Animal Blood Kit and reverse-transcribed, using the High Capacity cDNA Reverse Transcription Kit (Life Technologies) according the manufacturer’s protocol. The expression of the RNAs was analysed using TaqMan^®^ Gene Expression MasterMix (Applied Biosystems) and rodent TaqMan^®^ Gene Expression Assays for Gpnmb (Rn00591060_m1) and S100A9 (Rn00585879_m1) (Applied Biosystems). Results were normalized to the expression of Tmcc2 (Rn01759254_m1) which was identified as unchanged in the RNA sequencing experiment. The gene expression analysis was run on an SDS7900HT real time PCR system with a final amount of cDNA of 20ng per well; raw Ct values were calculated using the SDS software v2.4. RNAs with Ct values > 35/ undetermined were excluded. Fold-change of expression was calculated using the comparative Ct method (2^-ΔΔCt^).

#### Analysis of whole blood RNA sequencing data

Using STAR aligner version 2.3.0e, human sequencing reads were aligned to hg19 and rat reads to Rn5. Cufflinks-2.2.1 was used to derive RPKMs and read counts based on Ensembl 70 annotation for rat and human. Differential expression was calculated based on voom transformed counts using R version 3.0.1 and the Bioconductor package limma version 3.18.13. *P*-values were false discovery rate (FDR) adjusted by applying Benjamini-Hochberg correction.

Each Differential Gene Expression Score (DGE score) has been calculated according to

$$DGE\;score\;=\;-{log}_{10}(FDR)*|{log}_{2}(x_{mean\;treated})-{log}_{2}(x_{mean\;untreated})|$$

*x*_*mean_treated*_ and *x*_*mean_untreated*_ correspond to the mean group RPKM of treated and untreated samples, respectively. For the human study, the arithmetic mean value has been used.

Additional information on materials and methods can be found in as supporting information in S1 Text.

## Results

### Assessment of phospho-Thr^172^-AMPK in L6 myoblast and PBMC lysates

**Fig 1:**
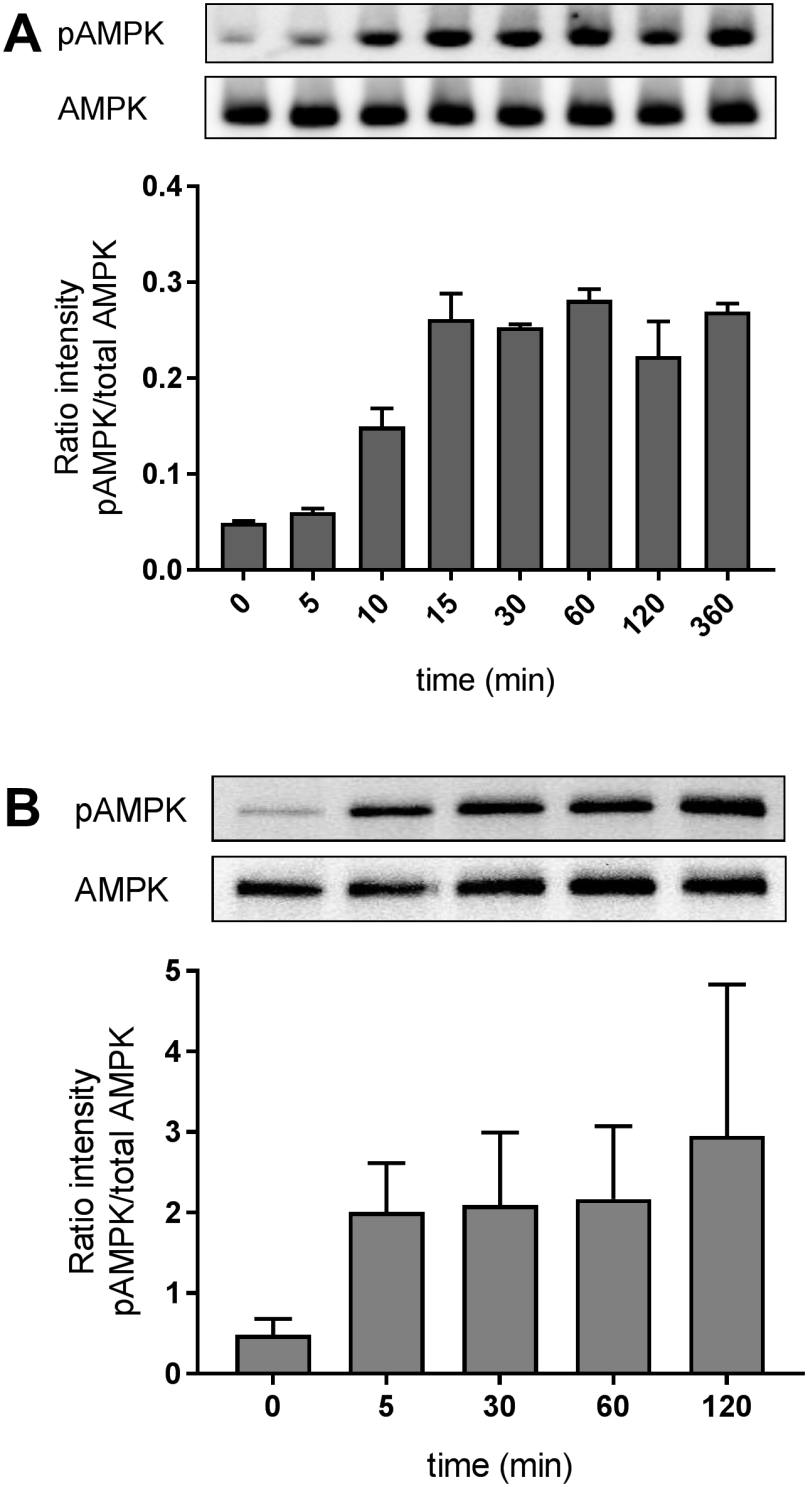
**A** AMPK Thr^172^-phosphorylation in L6 myoblast after stimulation with 1μM compound 2 and western blot densitometry of 3 independent experiments **B** AMPK phosphorylation in human PBMCs after stimulation with 10μM compound 2. Bars represent the mean +/- SEM of three independent experiments of the fluorescent analysis with stimulated PBMCs from different donors. Signals on western blot are representative for 2 donors and illustrated in false colors.

In order to assess the activity of a direct AMPK activator, L6 myoblast cells were incubated with 1μM compound 2 for up to 360 minutes and the phosphorylation of AMPK was analysed by Western blot. Activation of AMPK by compound 2 led to a 3-fold increase of the phospho-Thr^172^-AMPK/total AMPK ratio already after 10 min and reached a plateau after 15 min with an up to 5-fold increase (Fig 1A).

To evaluate whether AMPK phosphorylation can also be induced in human PBMCs, PBMCs were prepared from blood samples of three healthy volunteers and stimulated *ex vivo* for up to 120 minutes. Due to the plasma protein binding of the compound of approx. 80%, a 10-fold higher concentration was chosen compared to the cell culture experiment above (10μM compound 2, Fig 1B). The ratio of phospho-Thr^172^-AMPK/total AMPK was increased approximately 4-fold after 5 to 120 min stimulation. Hence, phosphorylation of AMPK was demonstrated *ex vivo* after stimulation in human PBMCs. Although AMPK phosphorylation is induced by compound 2 in human PBMCs the variability and the instability of the phosphorylation in general, make it unsuitable as a target engagement marker in the periphery in clinical studies. Therefore, we aimed for identification of a more stable and further downstream target engagement biomarker for AMPK activation using RNA sequencing.

### Biomarker discovery by RNA sequencing of human whole blood

In order to discover AMPK-regulated genes that may serve as blood based target engagement biomarkers, human whole blood from 4 healthy volunteers was incubated with compound 2 or DMSO for 6 hours. 608 genes were identified as significantly deregulated based on compound 2 treatment (FDR < 0.05; absolute FC > 2). Cathepsin D, interleukin 8, MAFF and LGALS3 were among the genes that were strongest upregulated based on compound 2 treatment (S1 Table). The genes with strongest repression were FFAR2, MNDA, RHOB, S100A8/A9 and CEACAM4 (S2 Table). In principle, these genes may be used as clinical target engagement biomarkers. However, we aimed to back-translate the sequencing results to pre-clinical animal models and to the diabetic setting as described in the following sections.

### Combined analysis of RNA sequencing results from human whole blood and HanWistar rat whole blood

In an effort to further identify translatable biomarker candidates, a subchronic animal study was performed in HanWistar rats treated with 30 mg compound 1 bid for 14 days. At the end of the study, blood samples were taken and mRNA Sequencing performed to identify AMPK-regulated genes. The results were compared with the results from the human whole-blood stimulation experiment.

In total, 367 rat genes mapping to known human genes were differentially expressed in treated vs. untreated rat blood samples (FDR < 0.05; FC >1.41) and 608 known human genes in *ex vivo* treated vs. untreated human blood samples (FDR < 0.05; FC > 2). The overlap from both studies is highly significant (p-value of the hypergeometric test < 0.001) and corresponds to 63 known human protein coding genes.

Further ranking of these 63 genes by robust expression in both studies (max RPKM≥ 5) and consistent regulation into the same direction predicts 22 genomic biomarker candidates including multiple secreted proteins. Table 1 shows these 22 genes ranked by the sum of the differential gene expression score (hereafter referred to as DGE score) for human and rat.

**Table 1:** Overlap of consistently AMPK-regulated genes in whole blood samples from HanWistar rats after subchronic treatment and from human after 6h *ex vivo* treatment. Genes have been ranked according to the total DGE score.

#	Gene Symbol	Recommended_Name	Regulation by AMPK activator	total DGE score	DGE score (rat)	DGE score (human)	Associated function	PMID
1	GPNMB	Transmembrane Glycoprotein Nmb	up	27,56	16,87	10,69	Downstream target of TFE3. AMPK regulates TFE3 directly or indirectly through FLCN/FNIP or under the regulation of FLCN/FNIP.	21209915
2	RHOB	Rho-Related Gtp-Binding Protein Rhob	down	14,98	3,21	11,77	Required for stability and nuclear trafficking of AKT1/AKT which promotes endothelial cell survival during vascular development.	UniProtKB
3	PGLYRP1	Peptidoglycan Recognition Protein 1	down	12,08	7,67	4,41	Mediates host defense against bacterial pathogens	22076558
4	S100A9	S100 Calcium Binding Protein A9	down	11,97	4,88	7,09	S100A8 and S100A9 are small calcium-binding proteins that are highly expressed in neutrophils and monocytes and chemotactic activities.	12626582
5	RENBP	Renin binding protein (N-Acylglucosamine 2-Epimerase)	up	11,75	2,88	8,87	Involved in inhibition of renin activity.	UniProtKB
6	S100A8	S100 Calcium Binding Protein A8	down	10,61	3,82	6,79	S100A8 and S100A9 are small calcium-binding proteins that are highly expressed in neutrophils and monocytes and chemotactic activities. Both respective proteins are biomarkers for CV disease.	12626582/ 18082488
7	RGS2	Regulator Of G-Protein Signaling 2	down	10,23	2,47	7,76	Plays a role in regulating the constriction and relaxation of vascular smooth muscle. Suppressed expression in prostate cancer cells after dominant-negative AMPK expression.	UniProtKB, 19347029
8	UBASH3B	Ubiquitin-Associated And Sh3 Domain-Containing Protein B	up	10,2	2,42	7,78	Was found to inhibit endocytosis of epidermal growth factor receptor (EGFR) and platelet-derived growth factor receptor.	Entrez Gene
9	UAP1L1	Udp-N-Acetylhexosamine Pyrophosphorylase-Like Protein 1	up	9,44	5,09	4,35	Involved in amino sugar and nucleotide sugar metabolism.	GeneCards
10	CXCR4	C-X-C Chemokine Receptor Type 4	up	9,23	3,33	5,9	Involved in immune response processes.	11276205
11	LRG1	Leucine-Rich Alpha-2-Glycoprotein	down	9,05	3,56	5,49	Involved in protein-protein interaction, signal transduction, and cell adhesion and development.	GeneCards
12	ANTXR2	Anthrax Toxin Receptor 2	down	7,99	3,68	4,31	Necessary for cellular interactions with laminin and the extracellular matrix.	UniProtKB
13	TMEM154	Transmembrane Protein 154	down	7,55	3,93	3,62	Unknown.	
14	PTAFR	Platelet-Activating Factor Receptor	down	6,95	2,64	4,31	Receptor for platelet activating factor, a chemotactic phospholipid mediator that possesses potent inflammatory, smooth-muscle contractile and hypotensive activity.	UniProtKB
15	CLEC4E	C-Type Lectin Domain Family 4 Member E	down	6,22	2,21	4	Involved in immune response processes.	Entrez Gene
16	AGPAT9	Glycerol-3-Phosphate Acyltransferase 3	down	6,13	3,1	3,03	Involved in glycerolipid biosynthesis. Regulated by AMPK in liver and adipose tissue in response to exercise,	12065578
17	MMP25	Matrix Metalloproteinase-25	down	5,62	2,07	3,55	Associated with degradation of extracellular matrix.	GeneCards
18	C1RL	Complement C1R Subcomponent-Like Protein	down	4,77	2,2	2,57	Involved in immune response processes.	Entrez Gene
19	CLEC4A	C-Type Lectin Domain Family 4 Member A	down	4,73	1,35	3,38	Involved in immune response processes.	Entrez Gene
20	NCF4	Neutrophil Cytosol Factor 4	down	4,72	1,29	3,43	Involved in immune response processes.	Entrez Gene
21	GABARAPL2	Gamma-Aminobutyric Acid Receptor-Associated Protein-Like 2	up	4,69	1,19	3,49	Modulates intra-Golgi transport through coupling between NSF activity and SNAREs activation and involved in autophagy.	UniProtKB
22	DHRS9	Dehydrogenase/Reductase Sdr Family Member 9	down	4,17	2,9	1,27	Converts 3-alpha-tetrahydroprogesterone to dihydroxyprogesterone and 3-alpha-androstanediol to dihydroxyprogesterone.	EntrezGene

As a promising starting point for biomarker development, this ranking predicts GPNMB as the strongest and most consistently regulated gene under these experimental conditions. GPNMB expression is increased by a factor of 4 in the treated vs. untreated rat and a factor of 19.7 in the treated vs. untreated human blood samples. In further experiments also RHOB, PGLYRP1 and S100A9 were evaluated as the most deregulated genes.

### Confirmation of gene candidates in concentration-response curves in human whole blood by Nanostring nCounter technology

**Fig 2:**
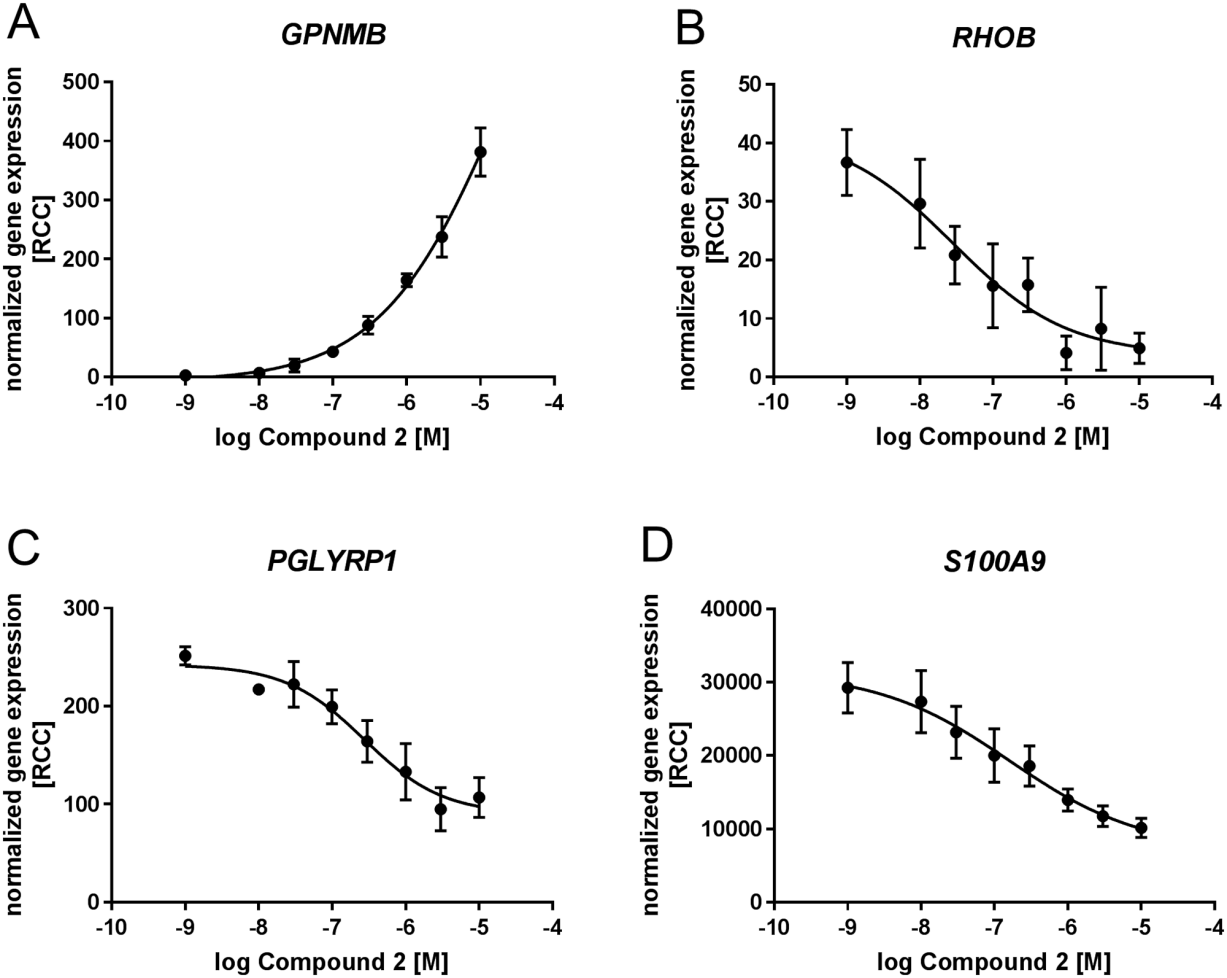
Concentration-response curves for compound 2 in human whole blood of 4 healthy volunteers. Expression values are normalized to the expression of 4 reference genes (ACTB, GAPDH, POL2RA, HPRT1). Normalized gene expression is expressed in reporter code counts (RCC) as mean ± SEM relative to DMSO-treated samples (10^−9^ value). Concentration-dependent regulation of **A** GPNMB **B** RHOB **C** PGLYRP1 and **D** S100A9.

To confirm that the identified translatable biomarker candidates GPNMB, RHOB, PGLYRP1 and S100A9 are regulated already at lower compound concentrations, blood samples from 4 healthy volunteers were stimulated *ex vivo* with increasing concentrations of compound 2. Gene expression was assessed using Nanostring nCounter technology. The expression of GPNMB was concentration-dependently increased by compound 2 with an up to 100-fold increase vs. the DMSO-treated control at 10μM compound 2. The expression of RHOB, PGLYRP1 and S100A9 was concentration-dependently decreased as expected (Fig 2). The most robust inhibition was seen with S100A9 and RHOB. However, RHOB expression was in general very low and therefore results were highly variable. PGLYRP1 expression was well detectable but only showed small changes in gene expression (approx. 2-fold inhibition). These results show that GPNMB, RHOB, PGLYRP1 and S100A9 are regulated in a concentration-dependent way by AMPK activation. Both, GPNMB and S100A9 provided the strongest changes in expression. Therefore, GPNMB and S100A9 were selected as most promising biomarker candidates to follow up in further experiments.

### GPNMB is specifically increased by AMPK activation

**Fig 3:**
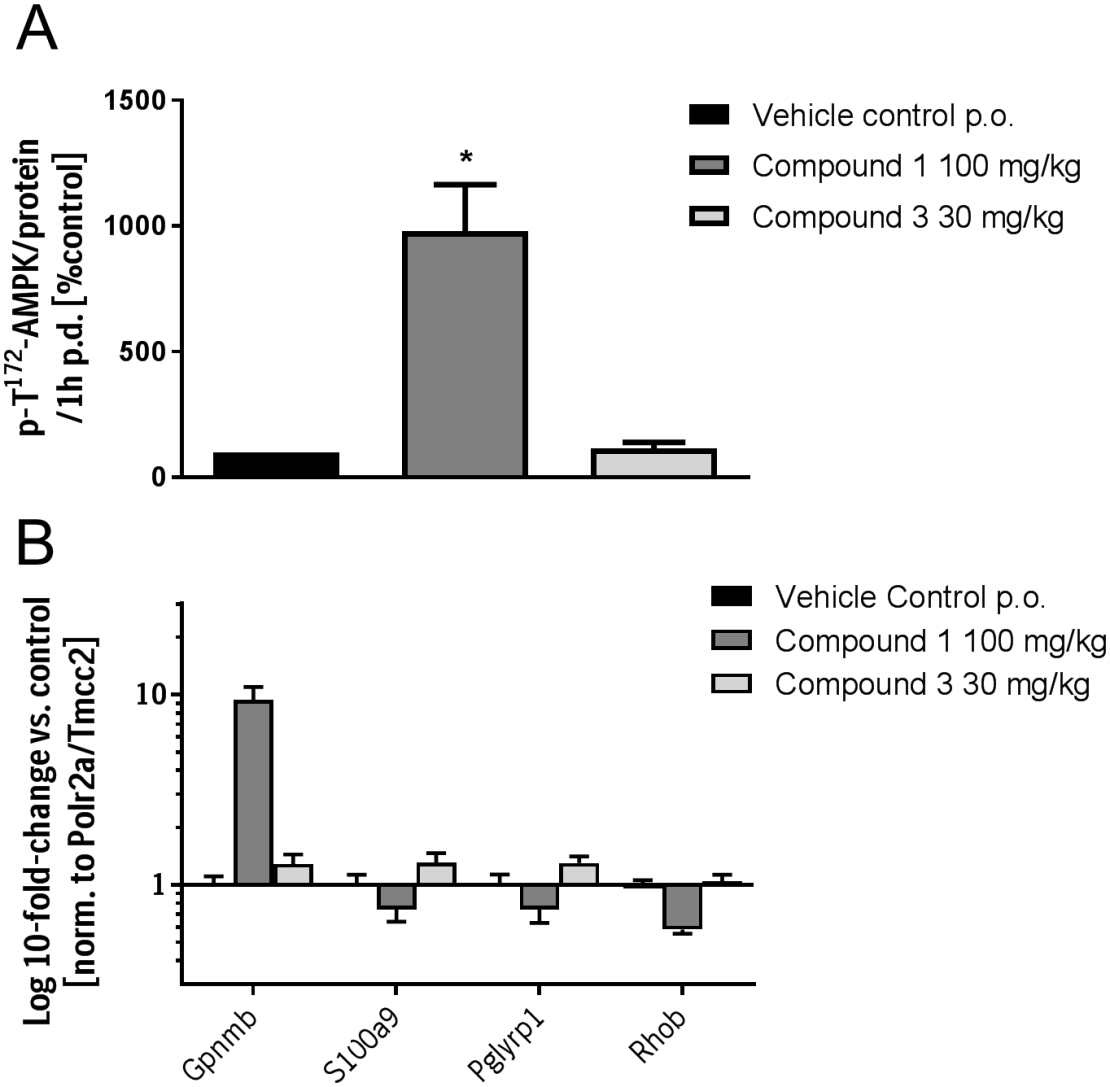
**A** AMPK Thr^172^-phosphorylation in liver 1h after dosing of HanWistar rats with the AMPK activator compound 1 and the inactive compound. **B** Gene expression of Gpnmb, S100A9, Pglyrp1 and RhoB. Bars represent the mean +/- SEM of five independent samples.

We aimed to confirm that GPNMB expression is specifically increased by AMPK activation. For this purpose, a structurally similar but inactive compound was tested (compound 3). Compound 3 did not induce the phosphorylation of AMPK at 1h post-dose in the liver of HanWistar rats in contrast to Compound 1 (Fig 3A). It could be shown that the inactive compound 3 did not induce GPNMB expression or reduced the expression of S100A9, PGLYRP1 or RHOB which indicates a specific regulation by AMPK (Fig 3B).

### Subchronic study in diabetic ZDF rats including target engagement in tissue and whole blood

**Fig 4:**
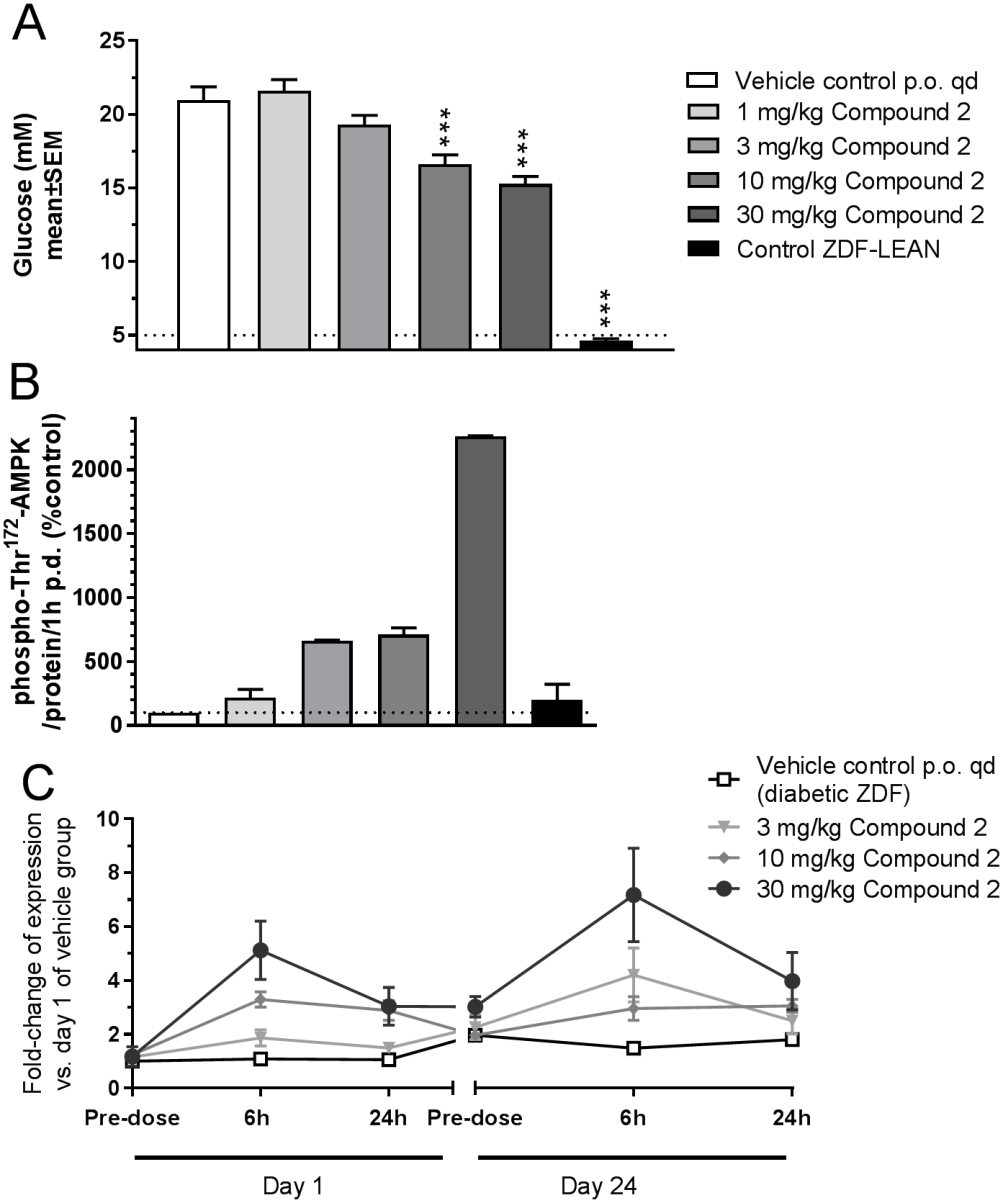
**A** Blood glucose level of male lean or diabetic ZDF rats (n = 10) after 28 days of dosing with vehicle or compound 2 (*** p<0.001 vs. Control) **B** Western blot densitometry of phospho-Thr^172^-AMPK (% of vehicle control) in liver tissue on day 30. **D** GPNMB expression in blood measured on day 1 and day 24 by qRT-PCR (Results are shown for 3–30 mg/kg doese).

After the discovery of potential target engagement markers in healthy individuals and normoglycemic HanWistar rats and confirmation of specificity to AMPK activation, we evaluated the regulation of GPNMB and S100A9 in diabetic ZDF rats as relevant disease model. After 28 days of treatment, in the vehicle-treated diabetic ZDF rats, blood glucose was 21.9±0.9 mM and significantly higher than in the lean ZDF rats (4.6±0.1 mM). In diabetic ZDF rats, compound 2 dose–dependently decreased blood glucose from 21.9±0.9 mM in the control group down to 16.6±0.6 mM at 10 mg/kg and to 15.3±0.5 mM at the dose of 30 mg/kg (Fig 4A). To show the activation of AMPK in tissues, phospho-T^172^-AMPK was measured in liver (Fig 4B) and skeletal muscle (data not shown). 30 days of treatment with compound 2 showed a dose-dependent increase in Thr^172^-AMPK phosphorylation between 6-fold (10 mg/kg) to 20-fold (30 mg/kg) in liver.

The expression of GPNMB was quantified in whole blood of ZDF rats using real-time PCR, after acute activation of AMPK on day 1 and after sub-chronic activation of AMPK on day 24, both 6h and 24h after dosing (Fig 4C). On day 1, in the 30 mg/kg group, AMPK activation led to an up to 5-fold increase in GPNMB expression after 6h versus pre-treatment values, with a small decline to 3-fold after 24h. On day 24, in the 30 mg/kg group, the pre-treatment value was already increased to approx. 3-fold of the day 1 pre-treatment value. After 6 h, however, there was a further increase of GPNMB expression of up to 7-fold compared to pre-treatment vehicle group) which again declined to approx. 4-fold at 24h after dosing. GPNMB expression in the control group showed small variability over all time points. The increase in GPNMB expression was dose-dependent (Fig 4C). In addition, the expression of S100A9 was reduced by compound 2 acutely and chronically, however the inhibition was only approx. 30–40% and not dose-dependent (data not shown). Therefore, S100A9 was not further investigated as translatable target engagement biomarker.

### Correlation of phospo-T172-AMPK in liver and muscle tissue to GPNMB increase in blood

**Fig 5:**
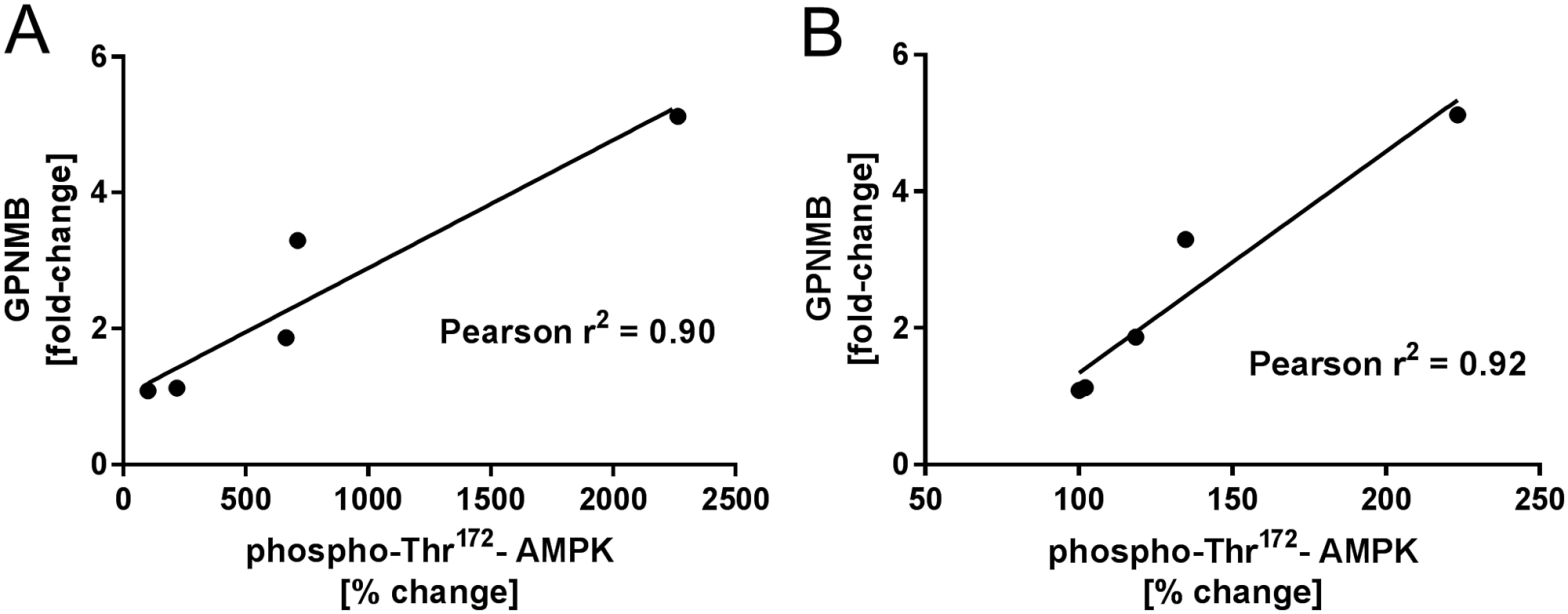
Correlation of GPNMB increase (Fold-change expression vs. day 1, vehicle group) in whole blood of ZDF rats with increase in phospho-Thr^172^-AMPK in two different tissues A Liver and B Quadriceps muscle.

Finally, the correlation of phospho-Thr^172^-AMPK activation in muscle and liver as the most important AMPK target tissues (day 30) to the fold-change in GPNMB expression in blood of ZDF rats (day 24) was assessed. A correlation could be shown for liver phospho-Thr^172^-AMPK (Pearson r = 0.90) and quadriceps muscle phospho-Thr^172^-AMPK (Pearson r = 0.92) (Fig 5). These results indicate that GPNMB expression in blood can be used as a surrogate biomarker for AMPK activation in liver and muscle tissue of diabetic ZDF rats.

## Discussion

The present study identified novel blood-based gene expression biomarkers, like GPNMB, which can be used as target engagement markers for future clinical trials with AMPK-activators. One specific aim was to identify biomarkers that could be back-translated to animal models and which show a correlation to tissue activation of AMPK in a disease model. GPNMB emerged as the most promising biomarker candidate showing a strong expression increase in whole blood from healthy volunteers and HanWistar rats as well as diabetic ZDF rats upon stimulation or treatment with an AMPK activator, respectively. In addition, GPNMB expression correlated well with the phosphorylation of AMPK in liver and skeletal muscle tissue. Therefore, GPNMB expression can be used as a surrogate marker for tissue AMPK activation in clinical studies.

A strength of the present study is the consistency of the GPNMB results across different models and time frames of stimulation/treatment e.g. *ex vivo* stimulation of human whole blood vs. 2-week treatment of normoglycemic HanWistar rats vs. 4-week treatment of diabetic ZDF rats. Another strength is compound 1 and compound 2 are potent and highly selective activators of AMPK, which minimizes the risk of identification of false-positive genes due to off-target effects of the compounds. For both compounds, only one non-selective inhibition of a kinase was identified in comprehensive selectivity testing (see Materials and methods). The common biological responses observed at low compound concentrations in this study could not be attributed to these two different kinases identified at 10 μM.

In addition, we have shown that compound 3 with structural similarities but no activity on AMPK did not increase GPNMB expression (Fig 3).

A potential weakness of the study is that we may have missed other potentially translatable biomarkers apart from GPNMB for two reasons A) because we did a pre-selection of translatable biomarkers based on the differential expression score which prioritized according to expression difference and significance level in rat and human and B) because we could only investigate a few genes in the subchronic study in ZDF rats due to volume limitations. Future studies should evaluate the value of other potentially translatable biomarkers from the lists provided in S1 and S2 Tables.

GPNMB seems well suited to serve as a peripheral target engagement marker as its increase in gene expression correlates well with the target tissue AMPK phosphorylation in ZDF rats. Future studies should make an attempt to model the increase of GPNMB (or other AMPK target engagement markers) in whole blood together with the tissue AMPK phosphorylation and the exposure of AMPK activators which might be useful for dose prediction in human studies. This approach may save future participants of clinical study the strain of muscle biopsies (at least in part). Future studies should investigate the clinical correlation between tissue AMPK activity, whole blood GPNMB expression and glucose lowering in diabetic patients. This aspect was beyond the scope of this enabling study. Additionally, future clinical studies should investigate whether AMPK activators also increase the level of soluble GPNMB protein in plasma.

GPNMB was discovered in melanoma cells as glycoprotein nonmetastatic melanoma B (GPNMB) and is also known as osteoactivin and dendritic cell-associated heparin sulfate proteoglycan-dependent integrin ligand [13–15]. It is an N-glycosylated type I transmembrane domain protein that is expressed in many cell types with high expression in dendritic cells, macrophages, liver, retinal pigment epithelial cells, osteoblasts and osteoclasts [13,14,16,17]. In macrophages, GPNMB is a feedback regulator of proinflammatory responses [18].

GPNMB expression has also been reported in breast cancer in more than 60% of tumors in stroma and in 10% in tumor epithelium [19]. GPNMB expression in tumor epithelium was an independent prognostic factor for breast cancer recurrence [19]. Additionally, GPNMB expression was most abundant in triple negative breast cancer and was a prognostic marker for shorter metastasis-free survival times within this breast cancer subtype. Functionally, GPNMB can promote cell migration, invasion and metastasis in breast cancer cells [19,20]. In this case, GPNMB is a prognostic biomarker, however, to date there a no reports that it has a causal role in breast cancer. In a meta-analysis that looked at breast cancer risk in type 2 diabetic patients taking metformin (an indirect AMPK activator) the OR was 0.83 in favor of metformin [21]. This speaks against a role of AMPK activation in breast cancer development.

Recently it was shown that transgenic overexpression of GPNMB ameliorated the fat accumulation and fibrosis in liver in a high fat, high sucrose fed obese mouse model [22]. In addition, GPNMB transgenic mice were protected from liver fibrosis under choline-deficient, L-amino acid-defined (CDAA) diet [23]. In a CCl4-induced acute liver injury, GPNMB expression was increased mostly in infiltrating CD68-positive macrophages, which are phagocytic macrophages with a role in repair processes of fibrotic liver [24].

Additionally, serum GPNMB protein level were found to be increased in type 2 diabetics compared to healthy controls and further increased in people with simple steatosis and with biopsy proven NASH [22]. The authors suggest soluble GPNMB as a novel marker for development and progression of NAFLD. Finally, current evidence suggests a protective role for GPNMB in hepatic fibrosis and a potential for soluble GPNMB. Therefore, when using GPNMB as a clinical PD biomarker for AMPK activation it is important to measure liver fibrosis markers in parallel because liver injury might be a confounding factor.

AMPK itself plays a major role in the control of hepatic metabolism by stimulating fatty acid oxidation and inhibition of lipogenesis, glucose production and protein synthesis [1,2]. Interestingly, metformin which act at least partially through AMPK but structurally unrelated to the compounds 1 and 2 was shown to induce the gene expression of GPNMB in primary rat hepatocytes from SD rats after incubation with 200μM metformin for 8 hours (Japanese Toxicogenomic Project, http://toxico.nibio.go.jp/english/index.html). Therefore, some of thebeneficial effects of AMPK activation on the liver might be mediated by GPNMB.

## Conclusion

In summary, we describe a novel potent and selective AMPK activator with glucose-lowering properties in a preclinical diabetes model. To advance AMPK activators into clinical studies, the translation and use of appropriate target engagement biomarkers is essential. Phospho-Thr^172^-AMPK assessment from PBMCs is not ideal in this regard as it has some limitations e.g. PBMC preparation at study site, instability of phosphorylated proteins and low-throughput western blotting procedures. We have identified GPNMB as a translatable indirect target engagement biomarker for use in clinical studies. The results also show the potential of RNA sequencing to identify novel target engagement biomarkers. Expression of GPNMB in whole blood of humans has several advantages over Phospho-Thr^172^-AMPK assessments in muscle e.g. accessibility of the matrix, sample stability and assay window. Due to the good correlation of whole blood GPNMB expression and Phospho-Thr^172^-AMPK in muscle and liver tissue upon AMPK activation, this relationship can be assumed for human studies and spares most study participants the strain of muscle biopsies. Recently, GPNMB was discussed as therapeutic target and biomarker for development and progression of NAFLD. Therefore, GPNMB could potentially link AMPK activation and fatty liver disease.

## Supporting information

S1 Table Top 10 genes with strongest increase upon compound 2-stimulation after 6 hours. (DOCX)

S2 Table Top 10 genes with strongest decrease upon compound 2-stimulation after 6 hours. (DOCX)

S3 Table Results of Panlabs Selectivity Screen of compound 1 (10μM) for 68 receptors. (DOCX)

S4 Table Results of Panlabs Selectivity Screen of compound 2 (10μM) for 68 receptors. (DOCX)

S5 Table Results of Invitrogen Selectivity Screen of compound 1 (10μM) for more than 260 kinases. (DOCX)

S6 Table Results of Invitrogen Selectivity Screen of compound 2 (10μM) for more than 260 kinases. (DOCX)

S1 Text Supplementary materials and methods. (DOCX)

## Abbreviations

ACTB: Actin beta
AMPK: AMP-activated protein kinase
CDAA: Choline-deficient, L-amino acid-defined diet
cDNA: Complementary DNA
DGE score: Differential Gene Expression Score
DMSO: Dimethyl sulfoxide
FDR: False discovery rate
GAPDH: Glycerinealdehyde 3-phosphate dehydrogenase
GPNMB: Transmembrane Glycoprotein Nmb
HPRT1: Hypoxanthine phosphoribosyltransferase 1
Log2FC: logarithm basis 2 of fold-change
NAFLD: Non-alcoholic fatty liver disease
NGS: Next Generation Sequencing
p.o. qd.: per oral, once daily administration
PBMC: Peripheral blood monocyte cells
PGLYRP1: Peptidoglycan recognition protein 1
POLR2A: RNA Polymerase II, subunit A
RCC: Relative reporter code counts
RHOB: Rho-related Gtp-binding protein RhoB
RNA: Ribonucleic acid
RPKM: Reads per kilobase per million mapped reds
S100A9: S100 Calcium-binding protein A9
SEM: Standard error of the mean
Thr^172^: Threonine 172 of AMPK
ZDF: Zucker diabetic fatty rats
