# Supplementary material for: Discovery and translation of a target engagement marker for AMP-activated protein kinase (AMPK)
Source: PLoS One. 2018 May 25;13(5):e0197849. doi: 10.1371/journal.pone.0197849 (PMC5969744; doi:10.1371/journal.pone.0197849)
Supplement: S1 Table — (DOCX) [file pone.0197849.s001.docx]

**S1 Table**: **Top 10 genes with strongest increase upon Compound 2-stimulation after 6 hours**. Whole-blood of blood from 4 healthy volunteers (according to the criteria: log2FC ≥ 1.5 & adj.p-value ≤ 0.01 & meanRPKM (DMSO) ≥ **5** & CV% ≤ 30).

| **#** | **Gene symbol** | **Recommended_Name** | **Compound 2 10µM 6h vs. DMSO 6h**  **Log2FC** |
| --- | --- | --- | --- |
| 1 | CTSD | cathepsin D | 3.307 |
| 2 | IL8 | interleukin 8 | 2.864 |
| 3 | PHACTR1 | phosphatase and actin regulator 1 | 2.840 |
| 4 | MAFF | v-maf musculoaponeurotic fibrosarcoma oncogene homolog F (avian) | 2.651 |
| 5 | ZNF438 | zinc finger protein 438 | 2.529 |
| 6 | LGALS3 | lectin, galactoside-binding, soluble, 3; galectin 3 | 2.495 |
| 7 | TBC1D2 | TBC1 domain family, member 2 | 2.461 |
| 8 | GNPDA1 | glucosamine-6-phosphate deaminase 1 | 2.389 |
| 9 | CD63 | CD63 molecule | 2.358 |
| 10 | MARCKS | myristoylated alanine-rich protein kinase C substrate | 2.349 |
