# Supplementary material for: Discovery and translation of a target engagement marker for AMP-activated protein kinase (AMPK)
Source: PLoS One. 2018 May 25;13(5):e0197849. doi: 10.1371/journal.pone.0197849 (PMC5969744; doi:10.1371/journal.pone.0197849)
Supplement: S2 Table — (DOCX) [file pone.0197849.s002.docx]

**S2 Table. Top 10 genes with strongest decrease** **upon Compound 2-stimulation after 6 hours.** Whole-blood stimulation of blood from 4 healthy volunteers (according to the criteria: log2FC ≤ -1.5 & adj.p-value ≤ 0.01 & meanRPKM (compound 2_6h) ≥ 5 & CV% ≤40 )

| **#** | **Gene symbol** | **Recommended_Name** | **Compound 2 10µM 6h vs. DMSO 6h**  **Log2FC** |
| --- | --- | --- | --- |
| 1 | FFAR2 | free fatty acid receptor 2 | -2.395 |
| 2 | MNDA | myeloid cell nuclear differentiation antigen | -2.017 |
| 3 | RHOB | ras homolog family member B | -1.967 |
| 4 | S100A8 | S100 calcium binding protein A8 | -1.837 |
| 5 | CEACAM4 | carcinoembryonic antigen-related cell adhesion molecule 4 | -1.791 |
| 6 | IMPA2 | inositol(myo)-1(or 4)-monophosphatase 2 | -1.759 |
| 7 | CPPED1 | calcineurin-like phosphoesterase domain containing 1 | -1.751 |
| 8 | S100A9 | S100 calcium binding protein A9 | -1.671 |
| 9 | ASF1B | ASF1 anti-silencing function 1 homolog B (S. cerevisiae) | -1.645 |
| 10 | CXCR2 | chemokine (C-X-C motif) receptor 2 | -1.602 |
