# Supplementary material for: Discovery and translation of a target engagement marker for AMP-activated protein kinase (AMPK)
Source: PLoS One. 2018 May 25;13(5):e0197849. doi: 10.1371/journal.pone.0197849 (PMC5969744; doi:10.1371/journal.pone.0197849)
Supplement: S4 Table — (DOCX) [file pone.0197849.s004.docx]

**S4 Table**: **Results of Panlabs Selectivity Screen of Compound 2 (10µM) for 68 receptors.**

| **Receptor**  **(PANLABS Method Name)** | **Compound 2**  **(% inhibition at 10µM)** |  | **Receptor**  **(PANLABS Method Name)** | **Compound 2**  **(% inhibition at 10µM)** |  | **Receptor**  **(PANLABS Method Name)** | **Compound 2**  **(% inhibition at 10µM)** |
| --- | --- | --- | --- | --- | --- | --- | --- |
| Thyroid Hormone | 5,0 |  | Muscarinic M3 | 15,0 |  | Transporter, Dopamine (DAT) | 3,0 |
| Sodium Channel, Site 2 | 23,0 |  | Muscarinic M2 | 9,0 |  | Dopamine D4.2 | 1,0 |
| Sigma σ1 | 5,0 |  | Muscarinic M1 | 6,0 |  | Dopamine D3 | 11,0 |
| Transporter, Serotonin (5- Hydroxytryptamine) (SERT) | 8,0 |  | Melatonin MT1 | -6,0 |  | Dopamine D2S | 6,0 |
| Serotonin (5-Hydroxytryptamine) 5-HT3 | 11,0 |  | Leukotriene, Cysteinyl CysLT1 | 17,0 |  | Dopamine D1 | 10,0 |
| Serotonin (5-Hydroxytryptamine) 5-HT2B | 6,0 |  | Interleukin IL-1 | 4,0 |  | Cannabinoid CB1 | 8,0 |
| Serotonin (5-Hydroxytryptamine) 5-HT1A | 10,0 |  | Imidazoline I2, Central | 7,0 |  | Calcium Channel N-Type | 0,0 |
| Rolipram | 26,0 |  | Histamine H3 | 10,0 |  | Calcium Channel L-Type, Dihydropyridine | 5,0 |
| Purinergic P2Y | 3,0 |  | Histamine H2 | 15,0 |  | Calcium Channel L-Type, Benzothiazepine | 15,0 |
| Purinergic P2X | 20,0 |  | Histamine H1 | 8,0 |  | Bradykinin B2 | 2,0 |
| Prostanoid EP4 | 15,0 |  | Glutamate, NMDA, Phencyclidine | 6,0 |  | Bradykinin B1 | 1,0 |
| Potassium Channel hERG | 4,0 |  | Glutamate, NMDA, Glycine | 6,0 |  | Androgen (Testosterone) | -3,0 |
| Potassium Channel [KATP] | -3,0 |  | Glutamate, NMDA, Agonism | -3,0 |  | Transporter, Norepinephrine (NET) | 9,0 |
| Platelet Activating Factor (PAF) | 2,0 |  | Glutamate, Kainate | 9,0 |  | Adrenergic β2 | 2,0 |
| Phorbol Ester | -3,0 |  | Glucocorticoid | 11,0 |  | Adrenergic β1 | -1,0 |
| Opiate μ(OP3, MOP) | 12,0 |  | GABAB1A | 13,0 |  | Adrenergic α2A | -5,0 |
| Opiate κ(OP2, KOP) | 11,0 |  | GABAA, Flunitrazepam, Central | -3,0 |  | Adrenergic α1D | 2,0 |
| Opiate δ1 (OP1, DOP) | 2,0 |  | GABAA, Muscimol, Central | 25,0 |  | Adrenergic α1B | 0,0 |
| Nicotinic Acetylcholine α1, Bungarotoxin | 12,0 |  | Transporter, GABA | -10,0 |  | Adrenergic α1A | -1,0 |
| Nicotinic Acetylcholine | 14,0 |  | Estrogen ERα | 20,0 |  | Adenosine A3 | -1,0 |
| Neuropeptide Y Y2 | 0,0 |  | Epidermal Growth Factor (EGF) | 4,0 |  | Adenosine A2A | 3,0 |
| Neuropeptide Y Y1 | 4,0 |  | Endothelin ETB | 0,0 |  | Adenosine A1 | 9,0 |
| Tachykinin NK1 | 14,0 |  | Endothelin ETA | 3,0 |  |  |  |
