# Supplementary material for: Discovery and translation of a target engagement marker for AMP-activated protein kinase (AMPK)
Source: PLoS One. 2018 May 25;13(5):e0197849. doi: 10.1371/journal.pone.0197849 (PMC5969744; doi:10.1371/journal.pone.0197849)
Supplement: S5 Table — (DOCX) [file pone.0197849.s005.docx]

**S5 Table.** **Results of Invitrogen Selectivity Screen of Compound 1 (10µM) for more than 260 Kinases.**

| **Protein Kinase** | **Compound 1**  **(% inhibition at 10µM)** |  | **Protein Kinase** | **Compound 1**  **(% inhibition at 10µM)** |  | **Protein Kinase** | **Compound 1**  **(% inhibition at 10µM)** |
| --- | --- | --- | --- | --- | --- | --- | --- |
| ZAP70 | -4,0 |  | MINK1 | -11,0 |  | CSNK1G1 | 11,0 |
| YES1 | 23,0 |  | MET M1250T | -3,0 |  | CSNK1E | 8,0 |
| TYRO3 | 8,0 |  | MET | 5,0 |  | CSNK1D | 2,0 |
| TYK2 | 11,0 |  | MERTK | 11,0 |  | CSNK1A1 | 2,0 |
| TXK | -3,0 |  | MELK | 21,0 |  | CSK | 4,0 |
| TEK | 19,0 |  | MATK | 1,0 |  | CSF1R | 19,0 |
| TBK1 | 0,0 |  | MARK4 | 6,0 |  | CLK3 | 3,0 |
| TAOK2 | -13,0 |  | INSR | -1,0 |  | CLK2 | 3,0 |
| SYK | 8,0 |  | IKBKE | 9,0 |  | CLK1 | 14,0 |
| STK4 | -12,0 |  | IKBKB | 23,0 |  | CHEK2 | -1,0 |
| STK3 | -23,0 |  | IGF1R | 5,0 |  | CHEK1 | -32,0 |
| STK25 | -12,0 |  | HIPK4 | 1,0 |  | CDK5/P35 | 9,0 |
| STK24 | -8,0 |  | HIPK3 | 1,0 |  | CDK5/P25 | 2,0 |
| STK23 | 1,0 |  | HIPK2 | 3,0 |  | CDK2/CYCLINA | -1,0 |
| STK22D | 3,0 |  | HIPK1 | 10,0 |  | CDK1/CYCLINB | 6,0 |
| STK22B | 8,0 |  | HCK | 7,0 |  | CDC42 BPB | -1,0 |
| SRPK2 | -13,0 |  | GSK3B | 14,0 |  | CDC42 BPA | -1,0 |
| SRPK1 | 2,0 |  | GSK3A | 8,0 |  | CAMK4 | -6,0 |
| SRMS | 8,0 |  | GRK7 | 6,0 |  | CAMK2D | 34,0 |
| SRC | 30,0 |  | GRK6 | 9,0 |  | CAMK2B | -1,0 |
| SRC N1 | 47,0 |  | GRK5 | 0,0 |  | CAMK2A | 16,0 |
| SNF1LK2 | -3,0 |  | GRK4 | 9,0 |  | CAMK1D | 0,0 |
| SGKL | 12,0 |  | FYN | 6,0 |  | BTK | 7,0 |
| SGK2 | 8,0 |  | FRK | 6,0 |  | BRSK1 | -4,0 |
| SGK | 7,0 |  | FRAP1 (MTOR) | -2,0 |  | BMX | 18,0 |
| RPS6KB1 | -3,0 |  | FLT4 | -3,0 |  | BLK | 3,0 |
| RPS6KA6 | 25,0 |  | FLT3 | 37,0 |  | AXL | -4,0 |
| RPS6KA5 | 0,0 |  | FLT1 | 1,0 |  | AURKC | -6,0 |
| RPS6KA4 | -5,0 |  | FGR | 24,0 |  | AURKB | 11,0 |
| RPS6KA3 | 16,0 |  | FGFR4 | 3,0 |  | STK6 | 18,0 |
| RPS6KA2 | -4,0 |  | FGFR3 | -9,0 |  | AMPK A2B1G1 | -210,0 |
| RPS6KA1 | 8,0 |  | FGFR3 K650E | -9,0 |  | AMPK A1B1G1 | -87,0 |
| ROS1 | 3,0 |  | FGFR2 | 2,0 |  | AKT3 | 31,0 |
| ROCK2 | -2,0 |  | FGFR1 | 3,0 |  | AKT2 | 13,0 |
| ROCK1 | 6,0 |  | FES | 35,0 |  | AKT1 | 8,0 |
| RET | 12,0 |  | FER | 15,0 |  | ADRBK2 | 5,0 |
| PTK6 | 3,0 |  | ERBB4 | 1,0 |  | ADRBK1 | 4,0 |
| PTK2B | 5,0 |  | ERBB2 | -14,0 |  | ACVR1B | -10,0 |
| PTK2 | 0,0 |  | EPHB4 | 11,0 |  | ABL2 | 8,0 |
| PRKX | 8,0 |  | EPHB3 | 3,0 |  | ABL1 | 11,0 |
| PRKG2 | 6,0 |  | EPHB2 | 13,0 |  | ZAK | -10,0 |
| PRKG1 | -4,0 |  | EPHB1 | 0,0 |  | WNK2 | -67,0 |
| PRKD2 | 0,0 |  | EPHA8 | 10,0 |  | WEE1 | -67,0 |
| PRKD1 | 7,0 |  | EPHA5 | 10,0 |  | TTK | -36,0 |
| PRKCZ | -5,0 |  | EPHA4 | 5,0 |  | TNK2 | 9,0 |
| PRKCQ | 35,0 |  | EPHA2 | 3,0 |  | TGFBR1 | 8,0 |
| PRKCN | 11,0 |  | EPHA1 | 8,0 |  | TEC | -22,0 |
| PRKCI | -7,0 |  | EGFR | -6,0 |  | TAOK3 | -93,0 |
| PRKCH | -14,0 |  | EEF2K | -3,0 |  | STK33 | -19,0 |
| PRKCG | 8,0 |  | DYRK4 | -3,0 |  | STK17A | -74,0 |
| PRKCE | 6,0 |  | DYRK3 | 17,0 |  | STK16 | -122,0 |
| PRKCD | -12,0 |  | DYRK1B | 3,0 |  | SLK | -6,0 |
| PRKCB2 | 13,0 |  | DYRK1A | -3,0 |  | RIPK2 | 20,0 |
| PRKCB1 | -11,0 |  | MARK3@CP | 4,0 |  | NLK | 16,0 |
| PRKCA | -3,0 |  | MARK2 | 6,0 |  | MYLK | 68,0 |
| PRKACA | -1,0 |  | MARK1 | -4,0 |  | MLCK | -32,0 |
| PLK3 | 40,0 |  | MAPKAPK5 | 6,0 |  | MKNK2 (MNK2) | -87,0 |
| PLK2 | 4,0 |  | MAPKAPK3 | 26,0 |  | MAPK9 | 12,0 |
| PLK1 | 21,0 |  | MAPKAPK2 | 27,0 |  | MAPK8 | -23,0 |
| PKN1 | -15,0 |  | MAPK3 | 3,0 |  | MAPK10 | 1,0 |
| PIM2 | 10,0 |  | MAPK14P38A | 0,0 |  | MAP3K7_K7IP1 | -44,0 |
| PIM1 | -3,0 |  | MAPK13 | 2,0 |  | MAP3K5 | -188,0 |
| PHKG2 | -1,0 |  | MAPK12 | 4,0 |  | MAP3K3 | -92,0 |
| PHKG1 | -4,0 |  | MAPK11 | 6,0 |  | MAP3K2 | -83,0 |
| PDK1DIRECT | -3,0 |  | MAPK1 | 4,0 |  | MAP3K14 | -60,0 |
| PDGFRB | 20,0 |  | MAP4K5 | 4,0 |  | MAP3K11 | -3,0 |
| PDGFRA | 13,0 |  | MAP4K4 | 1,0 |  | MAP3K10 | -11,0 |
| PASK | 0,0 |  | MAP4K2 | 9,0 |  | MAP2K6 | -22,0 |
| PAK7 | -6,0 |  | MAP3K9 | -2,0 |  | MAP2K6_S_T_E | -18,0 |
| PAK6 | -3,0 |  | LYN B | 10,0 |  | MAP2K3 | -297,0 |
| PAK4 | -17,0 |  | LYN A | 2,0 |  | MAP2K2 | -15,0 |
| PAK3 | 44,0 |  | LTK | 1,0 |  | MAP2K1 | -18,0 |
| PAK2 | 6,0 |  | LCK | 8,0 |  | LIMK2 | -2,0 |
| PAK1 | -15,0 |  | KIT | 12,0 |  | LIMK1 | -42,0 |
| NTRK3 | 6,0 |  | KDR | 6,0 |  | EPHA7 | -63,0 |
| NTRK2 | 0,0 |  | JAK3 | 16,0 |  | EPHA3 | -73,0 |
| NTRK1 | 28,0 |  | JAK2 | 11,0 |  | DMPK | -16,0 |
| NEK9 | -4,0 |  | JAK1 | -20,0 |  | DDR2 | -9,0 |
| NEK7 | -2,0 |  | ITK | 14,0 |  | DDR1 | 5,0 |
| NEK6 | 2,0 |  | IRAK4 | -7,0 |  | CLK4 | -45,0 |
| NEK4 | 4,0 |  | INSRR | -8,0 |  | CDK9_CYCLINK | -154,0 |
| NEK2 | -12,0 |  | DNA-PK | 5,0 |  | CDK8_CYCLINC | 9,0 |
| NEK1 | -10,0 |  | DCAMKL2 | 1,0 |  | CAMKK2 | 3,0 |
| MYLK2 | -5,0 |  | DAPK3 | 0,0 |  | CAMKK1 | -145,0 |
| MUSK | 22,0 |  | CSNK2A2 | 3,0 |  | BRAF | -27,0 |
| MST4 | -7,0 |  | CSNK2A1 | 11,0 |  | BMPR1A(ALK3) | -1,0 |
| MST1R | 6,0 |  | CSNK1G3 | 3,0 |  | ACVR2B | -12,0 |
| MKNK1 | -6,0 |  | CSNK1G2 | 8,0 |  | ACVR1 | -8,0 |
