# Supplementary material for: Discovery and translation of a target engagement marker for AMP-activated protein kinase (AMPK)
Source: PLoS One. 2018 May 25;13(5):e0197849. doi: 10.1371/journal.pone.0197849 (PMC5969744; doi:10.1371/journal.pone.0197849)
Supplement: S6 Table — (DOCX) [file pone.0197849.s006.docx]

**S6 Table. Results of Invitrogen Selectivity Screen of Compound 2 (10µM) for more than 260 Kinases.**

| **Protein Kinase** | **Compound 2**  **(% inhibition)** |  | **Protein Kinase** | **Compound 2**  **(% inhibition)** |  | **Protein Kinase** | **Compound 2**  **(% inhibition)** |
| --- | --- | --- | --- | --- | --- | --- | --- |
| SPHK1 | -19,0 |  | ADRBK2 | -2,0 |  | MARK2 | 9,0 |
| PIK3CG | 12,0 |  | ADRBK1 | 6,0 |  | MARK1 | 5,0 |
| PIK3CDPIK3R1 | 7,0 |  | ACVR1B | -3,0 |  | MAPKAPK5 | 8,0 |
| PIK3CAPIK3R1 | 7,0 |  | ABL2 | 5,0 |  | MAPKAPK3 | 11,0 |
| PIK3C3 | 5,0 |  | ABL1 | 9,0 |  | MAPKAPK2 | 13,0 |
| PIK3C2A | -7,0 |  | ZAP70 | 3,0 |  | MAPK3 | 0,0 |
| PI4KB | 3,0 |  | YES1 | 14,0 |  | MAPK14P38A | 8,0 |
| NUAK1 | -1,0 |  | TYRO3 | -3,0 |  | MAPK13 | 2,0 |
| LRRK2 | 9,0 |  | TYK2 | 4,0 |  | MAPK12 | 2,0 |
| LRRK2_FL | 8,0 |  | TXK | 10,0 |  | MAPK11 | -1,0 |
| IRAK1 | -20,0 |  | TEK | 15,0 |  | MAPK1 | 8,0 |
| GSG2 | -4,0 |  | TBK1 | 3,0 |  | MAP4K5 | 2,0 |
| DAPK1 | 15,0 |  | TAOK2 | -9,0 |  | MAP4K4 | -26,0 |
| CHUK_IKKA | 2,0 |  | SYK | 5,0 |  | MAP4K2 | -1,0 |
| CDK9CYCLIN | -4,0 |  | STK4 | 7,0 |  | MAP3K9 | -12,0 |
| CDK7CYCLIN | -7,0 |  | STK3 | 7,0 |  | LYN B | 5,0 |
| IGF1R | 7,0 |  | STK25 | -3,0 |  | LYN A | 6,0 |
| HIPK4 | 4,0 |  | STK24 | -10,0 |  | LTK | -2,0 |
| HIPK3 | 2,0 |  | STK23 | 6,0 |  | LCK | -5,0 |
| HIPK2 | 9,0 |  | STK22D | 3,0 |  | KIT | -9,0 |
| HIPK1 | 3,0 |  | STK22B | 7,0 |  | KDR | 8,0 |
| HCK | 10,0 |  | SRPK2 | -2,0 |  | JAK3 | 32,0 |
| GSK3B | 6,0 |  | SRPK1 | -8,0 |  | JAK2 | -19,0 |
| GSK3A | 0,0 |  | SRMS | 18,0 |  | JAK1 | -16,0 |
| GRK7 | -2,0 |  | SRC | 9,0 |  | ITK | 2,0 |
| GRK6 | -2,0 |  | SRC N1 | 6,0 |  | IRAK4 | -5,0 |
| GRK5 | 3,0 |  | SNF1LK2 | 1,0 |  | INSRR | 3,0 |
| GRK4 | 5,0 |  | SGKL | -3,0 |  | INSR | 0,0 |
| FYN | 6,0 |  | SGK2 | 1,0 |  | IKBKE | 6,0 |
| FRK | 8,0 |  | SGK | -13,0 |  | IKBKB | 2,0 |
| FRAP1 (MTOR) | 3,0 |  | RPS6KB1 | -8,0 |  | ZAK | 8,0 |
| FLT4 | 3,0 |  | RPS6KA6 | 12,0 |  | WNK2 | 6,0 |
| FLT3 | 35,0 |  | RPS6KA5 | -1,0 |  | WEE1 | 7,0 |
| FLT1 | 11,0 |  | RPS6KA4 | 7,0 |  | TTK | -3,0 |
| FGR | 25,0 |  | RPS6KA3 | 6,0 |  | TNK2 | 6,0 |
| FGFR4 | 1,0 |  | RPS6KA2 | 2,0 |  | TGFBR1 | 2,0 |
| FGFR3 | 2,0 |  | RPS6KA1 | 2,0 |  | TEC | -2,0 |
| FGFR3 K650E | 4,0 |  | ROS1 | -2,0 |  | TAOK3 | 7,0 |
| FGFR2 | 6,0 |  | ROCK2 | 1,0 |  | STK33 | 1,0 |
| FGFR1 | 2,0 |  | ROCK1 | -13,0 |  | STK17A | 5,0 |
| FES | 9,0 |  | RET | 10,0 |  | STK16 | -12,0 |
| FER | 2,0 |  | PTK6 | 6,0 |  | SLK | -1,0 |
| ERBB4 | -7,0 |  | PTK2B | 1,0 |  | RIPK2 | 2,0 |
| ERBB2 | -1,0 |  | PTK2 | 3,0 |  | NLK | 12,0 |
| EPHB4 | 6,0 |  | PRKX | 2,0 |  | MYLK | 97,0 |
| EPHB3 | 9,0 |  | PRKG2 | 4,0 |  | MLCK | 9,0 |
| EPHB2 | -2,0 |  | PRKG1 | 2,0 |  | MKNK2 (MNK2) | 8,0 |
| EPHB1 | 3,0 |  | PRKD2 | 9,0 |  | MAPK9 | -7,0 |
| EPHA8 | 3,0 |  | PRKD1 | 11,0 |  | MAPK8 | -4,0 |
| EPHA5 | 9,0 |  | PRKCZ | -5,0 |  | MAPK10 | -1,0 |
| EPHA4 | 14,0 |  | PRKCQ | 16,0 |  | MAP3K7_K7IP1 | -3,0 |
| EPHA2 | -1,0 |  | PRKCN | 14,0 |  | MAP3K5 | 3,0 |
| EPHA1 | 8,0 |  | PRKCI | -6,0 |  | MAP3K3 | 3,0 |
| EGFR | 9,0 |  | PRKCH | 4,0 |  | MAP3K2 | -1,0 |
| EEF2K | 2,0 |  | PRKCG | 9,0 |  | MAP3K14 | 7,0 |
| DYRK4 | 0,0 |  | PRKCE | -6,0 |  | MAP3K11 | 2,0 |
| DYRK3 | 1,0 |  | PRKCD | -6,0 |  | MAP3K10 | 0,0 |
| DYRK1B | -1,0 |  | PRKCB2 | 0,0 |  | MAP2K6 | 0,0 |
| DYRK1A | 1,0 |  | PRKCB1 | 6,0 |  | MAP2K6_S_T_E | 1,0 |
| DNA-PK | 7,0 |  | PRKCA | 10,0 |  | MAP2K3 | -2,0 |
| DCAMKL2 | 5,0 |  | PRKACA | -1,0 |  | MAP2K2 | -1,0 |
| DAPK3 | -17,0 |  | PLK3 | 4,0 |  | MAP2K1 | -3,0 |
| CSNK2A2 | 6,0 |  | PLK2 | 8,0 |  | LIMK2 | 9,0 |
| CSNK2A1 | 3,0 |  | PLK1 | 4,0 |  | LIMK1 | 25,0 |
| CSNK1G3 | 5,0 |  | PKN1 | 1,0 |  | EPHA7 | 3,0 |
| CSNK1G2 | -5,0 |  | PIM2 | 2,0 |  | EPHA3 | 6,0 |
| CSNK1G1 | 4,0 |  | PIM1 | 0,0 |  | DMPK | 0,0 |
| CSNK1E | 12,0 |  | PHKG2 | -6,0 |  | DDR2 | 0,0 |
| CSNK1D | 0,0 |  | PHKG1 | -6,0 |  | DDR1 | 3,0 |
| CSNK1A1 | 3,0 |  | PDK1DIRECT | -2,0 |  | CLK4 | 1,0 |
| CSK | 5,0 |  | PDGFRB | 25,0 |  | CDK9_CYCLINK | -1,0 |
| CSF1R | 11,0 |  | PDGFRA | -6,0 |  | CDK8_CYCLINC | 0,0 |
| CLK3 | 2,0 |  | PASK | 5,0 |  | CAMKK2 | 9,0 |
| CLK2 | 4,0 |  | PAK7 | -2,0 |  | CAMKK1 | 5,0 |
| CLK1 | 8,0 |  | PAK6 | -8,0 |  | BRAF | -2,0 |
| CHEK2 | -1,0 |  | PAK4 | -2,0 |  | BMPR1A(ALK3) | 2,0 |
| CHEK1 | -7,0 |  | PAK3 | 4,0 |  | ACVR2B | 0,0 |
| CDK5/P35 | 25,0 |  | PAK2 | 17,0 |  | ACVR1 | 5,0 |
| CDK5/P25 | -2,0 |  | PAK1 | -36,0 |  | AKT3 | 5,0 |
| CDK2/CYCLINA | 2,0 |  | NTRK3 | 4,0 |  | AKT2 | 10,0 |
| CDK1/CYCLINB | 5,0 |  | NTRK2 | 27,0 |  | AKT1 | 0,0 |
| CDC42 BPB | -16,0 |  | NTRK1 | 4,0 |  | MATK | -6,0 |
| CDC42 BPA | -16,0 |  | NEK9 | -7,0 |  | MARK4 | -7,0 |
| CAMK4 | -10,0 |  | NEK7 | -2,0 |  | MARK3@CP | 2,0 |
| CAMK2D | 7,0 |  | NEK6 | 6,0 |  |  |  |
| CAMK2B | 6,0 |  | NEK4 | 2,0 |  |  |  |
| CAMK2A | 4,0 |  | NEK2 | -10,0 |  |  |  |
| CAMK1D | 5,0 |  | NEK1 | -13,0 |  |  |  |
| BTK | 3,0 |  | MYLK2 | -12,0 |  |  |  |
| BRSK1 | -10,0 |  | MUSK | 1,0 |  |  |  |
| BMX | 7,0 |  | MST4 | -17,0 |  |  |  |
| BLK | 7,0 |  | MST1R | 7,0 |  |  |  |
| AXL | 5,0 |  | MKNK1 | 16,0 |  |  |  |
| AURKC | -3,0 |  | MINK1 | -81,0 |  |  |  |
| AURKB | 3,0 |  | MET M1250T | 3,0 |  |  |  |
| STK6 | 3,0 |  | MET | 4,0 |  |  |  |
| AMPK A2B1G1 | -412,0 |  | MERTK | 18,0 |  |  |  |
| AMPK A1B1G1 | -53,0 |  | MELK | 13,0 |  |  |  |
