# Supplementary material for: Discovery and translation of a target engagement marker for AMP-activated protein kinase (AMPK)
Source: PLoS One. 2018 May 25;13(5):e0197849. doi: 10.1371/journal.pone.0197849 (PMC5969744; doi:10.1371/journal.pone.0197849)
Supplement: S1 Text — (DOCX) [file pone.0197849.s007.docx]

**S7 Methods. Supplementary materials and methods.**

**AMPK Activation Assay in L6 myoblast cells**

The activation of AMPK was determined by quantification of Thr^172^-phosphorylated AMPK. L6 myoblasts were treated with 1 µM compound 2 in alphaMEM. At various times, cells were lysed in lysis buffer (50Mm Tris-HCL pH7.4, 150mM NaCl, 1mM EGTA, 10mM EDTA, 1% Triton-X 100, 0.1% SDS, 0.5% DeoxycholicAcid, 1% NP-40, 10mM Beta-glycerophosphate, 10mM Natriumpyrophosphate and 1x Complete EDTA free Protease Inhibitor). Proteins were separated by SDS PAGE (E-Page System Invitrogen), transferred onto nitrocellulose membranes (iBlot System, Invitrogen) and detected by using anti-phospho AMPK antibody, (dilution1:1000, Cat # 5256, Cell Signaling) as primary and anti-rabbit IgG HRP conjugate, (dilution1:10000, Cat # W4011, Promega) as secondary antibody. Signal was detected using Fuji LAS 3000 Imaging station (Fuji Photo Film; Fuji, Tokyo, Japan) and quantified using AIDA Image Analyser software (Raytest, Straubenhardt, Germany). The ratio between Phospho-Thr^172^-AMPK and AMPK was calculated.

**Analysis of phospho-AMPK from human PBMCs**

The activation of AMPK was determined by quantification of Thr^172^-phosporylated AMPKα and AMPKα in human PBMC lysates. PBMCs (peripheral blood mononuclear cells) were extracted from Sodium-Heparin blood by using Leukosep tubes with Histopaque (Leukosep Tube, Cat. # 163289, Greiner ; Histopaque, Cat. # 1077, Sigma). The PBMCs were washed with PBS, resuspended in RPMI 1640 medium with L-Glutamine (Cat. # 11875-093, GIBCO) and treated with 10 µM of compound 2 at a concentration of 0.1 % DMSO and 37°C. At the timepoints 0 (=Baseline), 5 min, 30 min, 60 min and 120 min the incubation was stopped by centrifugation, followed by a washing step with ice cold PBS. The PBMC pellets were lysed with lysis buffer for 15 minutes on ice (TRIS-Lysis buffer, Cat. # 61870-010, MSD; 1x Protease/Phosphatase inhibitor Mix, Cat. # 1861281, Thermo Scientific; 1 mM Sodium Orthovanadate, Cat. # P0758S, BioLabs). The lysates were diluted with 2 x Lämmli Sample buffer and incubated at 95°C for 5 minutes. The proteins were separated by SDS PAGE (10% Mini-Protean TGX Stain Free Gels, Cat # 456-8033, BIORD; TRIS/Glycin/SDS-Buffer, Cat # 161-0732, BIORAD) and transferred onto PVDF membranes (Mini-size LF PVDF membranes, Cat.#170-4274; TransBlot Trubo, BIORAD). The phospho-Thr^172^-AMPKα and AMPKα detection was conducted on two separated blots by using either phospho-AMPKα (Thr172) antibody (dilution 1:250, Cat # 2535, Cell Signaling) or AMPKα (dilution 1:1000, Cat # 2532, Cell Signaling) as primary antibody and anti-rabbit IgG-AF647 conjugate (dilution 1:1000, Cat # 4414, Cell Signaling) as secondary antibody. Signals were recorded using ChemiDoc MP Imaging System (BIORAD) and quantified using Image Lab software (BIORAD, Version 5.0). The signal for AF647 was normalized by the stain-free blot volume integral to exclude loading artefacts. The phosphorylation status of PBMC´s was expressed as phospho-Thr^172^-AMPK/AMPK ratio.

**Whole blood stimulation and RNA-Sequencing**

Whole blood from four healthy volunteers was investigated. The blood was collected in BD Vacutainer^®^ EDTA tubes (Becton, Dickinson and Company, Franklin Lakes, New Jersey, U.S.A.) and stimulated for 6 hours at 37°C with compound 2 with a final concentration of 10µM. After incubation, 2.5 mL stimulated whole blood was transferred into PAXgene RNA tubes (PreAnalytiX GmbH, Hombrechtikon, Switzerland). Tubes were stored frozen and thawed for extraction process at room temperature (RT) over night. RNA extraction was performed using the PAXgene Blood RNA Kit (Qiagen, Hilden, Germany) according to the vendor’s instructions.

Before starting NGS workflow a globin depletion was performed with 14 µL extracted RNA using human GLOBINclear^TM^ Kit (Ambion by Thermo Fisher Scientific, Waltham, MA U.S.A.) according to the manufacturer’s protocol. Quantity of globin-depleted total RNA was determined by absorbance measurement using a NanoDrop Spectrophotometer. Samples were normalized to 4 ng/µL. For manual sample preparation TruSeq RNA Sample Preparation Kit v2 - Set B (Illumina^®^, [San Diego, U.S.A](https://www.google.de/search?q=San+Diego&stick=H4sIAAAAAAAAAOPgE-LSz9U3MDIvMUxPUeIAsc0Ny4q0tLKTrfTzi9IT8zKrEksy8_NQOFYZqYkphaWJRSWpRcUANbq9m0QAAAA&sa=X&ved=0ahUKEwjRh8GptNHMAhVRKywKHUEAB6UQmxMIoQEoATAQ).) was used with a starting amount of 200ng total RNA according to the manufacturer’s instructions. Library quantity and quality was determined using Quant-it Pico Green dsDNA reagent (Invitrogen by Thermo Fisher Scientific, Waltham, MA U.S.A.) via a microplate reader (BMG labtech, Ortenberg, Germany) and by checking cDNA fragment size of about 10% of total sample number processed using a DNA1000 Kit (Agilent Technologies Inc, Waldbronn, Germany) on the Agilent Bioanalyzer 2100 (Agilent Technologies). Library stocks were diluted to 2nM and samples with different indexes were pooled.

Automated cluster generation was performed on a Illumina^®^ cBot system driven by cBot software (TruSeq SR Cluster Kit v3 – cBot – HS, Illumina^®^) and Sequencing was run on a Illumina^®^ HiSeq 2000 system driven by HiSeq Control software (TruSeq SBS Kit HS- v3 (52+7 cycles, single-read); Illumina^®^).

**Selectivity Tests**

**Receptor Panel:**

Radioligand Binding Assay at Eurofins Panlabs, Taiwan employed in this study have been adapted from the scientific literature to maximize reliability and reproducibility of each individual assay. Reference standards were run as an integral part of each assay to ensure the validity of the results obtained.

**Kinase Panel:**

Compounds were tested in the Lantha Screen Kinase Activity Assay or the Z´Lyte Assay format at ThermoFisher, Scientific/Invitrogen.
